# Body size is more important than diet in determining stable-isotope estimates of trophic position in crocodilians

**DOI:** 10.1038/s41598-018-19918-6

**Published:** 2018-01-31

**Authors:** Francisco Villamarín, Timothy D. Jardine, Stuart E. Bunn, Boris Marioni, William E. Magnusson

**Affiliations:** 1 Coordenação de Pesquisas em Biodiversidade, Instituto Nacional de Pesquisas da Amazônia - INPA, Manaus, Brazil; 2 Universidad Regional Amazónica - Ikiam, Tena, Ecuador; 3 School of Environment and Sustainability, University of Saskatchewan, Saskatoon, Canada; 4 Australian Rivers Institute, Griffith University, Brisbane, Australia; 5 Programa de Pós-Graduação em Biologia de Água Doce e Pesca Interior - INPA, Manaus, Brazil

## Abstract

The trophic position of a top predator, synonymous with food-chain length, is one of the most fundamental attributes of ecosystems. Stable isotope ratios of nitrogen (*δ*^15^N) have been used to estimate trophic position of organisms due to the predictable enrichment of ^15^N in consumer tissues relative to their diet. Previous studies in crocodilians have found upward ontogenetic shifts in their ‘trophic position’. However, such increases are not expected from what is known about crocodilian diets because ontogenetic shifts in diet relate to taxonomic categories of prey rather than shifts to prey from higher trophic levels. When we analysed dietary information from the literature on the four Amazonian crocodilians, ontogenetic shifts in dietary-based trophic position (TP_diet_) were minimal, and differed from those estimated using *δ*^15^N data (TP_SIA_). Thus, ontogenetic shifts in TP_SIA_ may result not only from dietary assimilation but also from trophic discrimination factors (TDF or *Δ* ^15^N) associated with body size. Using a unique TDF value to estimate trophic position of crocodilians of all sizes might obscure conclusions about ontogenetic shifts in trophic position. Our findings may change the way that researchers estimate trophic position of organisms that show orders of magnitude differences in size across their life span.

## Introduction

The trophic position of a top predator is an important component of food-web structure because it reflects the number of steps that energy takes to reach it from basal resources. This is synonymous with food-chain length, which is considered to be one of the most fundamental attributes of ecosystems^1–3^. During recent decades, stable isotope ratios of nitrogen (*δ* ^15^N) have been widely used to estimate trophic position of organisms as a continuous measure^4^, and this use has become the standard for most food web studies. The use of *δ* ^15^N as a surrogate of trophic position is based on the knowledge that the tissues of consumers become ^15^N-enriched relative to their diets^5–7^. The mechanism underlying this pattern has been traditionally thought to be related to the higher rate of excretion of light isotopes (^14^N) in relation to heavy isotopes (^15^N), a process that leads to isotopic discrimination^6^. More recently, isotopic discrimination has been proposed to occur both during assimilation and protein synthesis and during the excretion of endogenous nitrogen in urine^8–10^, which would indicate a possible effect of metabolic efficiency on TDF values. TDF values range widely across the animal kingdom^4^, with average values between 2.0^11^ and 3.4‰^6^ for most of the studied organisms (summarized in ref.^12^). However, crocodilians show a much wider range of TDF values than other vertebrates^13–16^.

As opportunistic predators^17,18^, crocodilians may strongly influence the structure of food webs in distinct ways by preying upon organisms occupying different trophic levels across aquatic and terrestrial systems^14,16,19–22^. In all species, crocodilians grow several orders of magnitude both in length and mass during their lifespan. Such marked shifts in size are likely to be associated with decreased metabolic and growth rates^23^, which strongly influence biochemical reactions and processes in the body, including protein turnover and excretion rates^24^. Therefore, it is plausible that the mechanisms leading to isotopic discrimination may undergo ontogenetic changes as well.

Another important implication of changes in body size is that crocodilians experience ontogenetic shifts in diet, eating terrestrial and aquatic invertebrates when young and larger prey composed mostly of vertebrates as they grow larger^25–28^. Amazonian crocodilians within the family Alligatoridae show this ontogenetic variation but exhibit interspecific differences in diet as adults. For example, adult *Paleosuchus palpebrosus* (Cuvier’s dwarf caiman), *Caiman crocodilus* (spectacled caiman) and *Melanosuchus niger* (black caiman), which attain up to 1.0, 1.4 and 2.5 m snout-vent length (SVL), respectively^29–31^, have diets mostly consisting of fish^28,32^. In contrast, adult *Paleosuchus trigonatus* (Schneider’s dwarf caiman), which can attain SVL of over 1.0 m^33^, consume terrestrial vertebrates and few fish^28^. It is uncertain how these dietary differences are reflected in *δ* ^15^N values within and among species, and the trophic position that Amazon crocodilians occupy.

Previous studies that analyzed *δ* ^15^N values in crocodilians, including individuals of broad size ranges, found ontogenetic increases in *δ* ^15^N values that were considered to mirror changes in trophic position caused exclusively by dietary shifts^14,34,35^. Although most of those studies included information on stomach-content analyses, none explicitly estimated the proportional contribution of each prey category to the diet of crocodilians of different sizes, which would have allowed direct estimation of prey trophic levels. This is crucial, because a shift from a diet mainly composed of invertebrates to a diet composed of vertebrates would not necessarily cause a linear increase in trophic position. Many invertebrates eaten by small crocodilians, such as Mygalomorph spiders are predators from high trophic levels, and vertebrates preyed upon by large crocodilians, such as fish and ground-dwelling mammals, may occupy low trophic levels. In fact, some are strictly herbivorous. Thus, known ontogenetic shifts in crocodilian diets do not indicate increasing amounts of prey from higher trophic levels despite shifts to different taxonomic categories of prey. If prey trophic position were the main determinant of isotopic shifts in crocodilian tissues, ontogenetic dietary trends would be expected to match isotopic shifts in crocodilian tissues. Uncoupled trends between dietary and isotopic data^36^ would suggest that ontogenetic shifts in trophic position of crocodilians may result from mechanisms additional to dietary assimilation. Growth rate is a proxy for metabolic rate since mass adjusted values for both decline with age^37,38^ and prior work has shown that growth rate can influence *δ* ^15^N discrimination in laboratory studies^39^. Thus, we might expect discrimination to change in concert with slowing growth and metabolic rates as crocodilians age. However, the magnitude of this effect, and whether it occurs under natural conditions is unknown.

Here, we explore *δ* ^15^N values in a broad size range of the four species of crocodilians occurring in the Amazon basin and relate this to literature information^28^ on the proportional contributions of prey from different trophic levels to the diet of different-sized crocodilians. By doing so, we evaluate the extent to which dietary changes are coupled with shifts in *δ* ^15^N values. We also estimated growth rates of the four species based on their size, using equations published in the literature^40–43^ in order to elucidate other possible mechanisms responsible for ontogenetic shifts in *δ* ^15^N values. Using those sources of evidence, we aim to determine to what extent ontogenetic shifts in diet are coupled with changes in *δ* ^15^N values of tissues of Amazonian crocodilians and whether size-related metabolic changes can explain part of those changes.

## Results

### Isotopic characterization of baseline organisms and crocodilians

Primary consumers had values that were similar in terrestrial (*Dasyprocta* sp. and *Cuniculus* sp., mean ± SD *δ* ^15^N values of 4.6 ± 1.9‰) and aquatic (*Pomacea* sp. snails; 3.8 ± 0.5‰) habitats (T-test; t = −1.67; df = 1.1; p = 0.327). We analyzed 45 *P*. *trigonatus* individuals between 23.8 and 99.2 cm SVL (*δ* ^15^N = 8.1 ± 0.9‰); 31 *C*. *crocodilus* individuals (30.3 to 97.8 cm SVL; *δ* ^15^N = 7.9 ± 1.1‰), 37 *P*. *palpebrosus* individuals (17.4 to 96.4 cm SVL; *δ* ^15^N = 8.1 ± 1.0‰) and 9 *M*. *niger* individuals (56.5 to 105.5 cm SVL; *δ* ^15^N = 6.8 ± 1.9‰). Mean trophic position assessed using nitrogen stable isotopes (TP_SIA_) ranged from 3.29 in *M*. *niger* to 3.97 in *P*. *trigonatus*.

### Ontogenetic shifts in TP_SIA_ as a function of body size, TP_diet_ and growth rates

**Figure 1:**
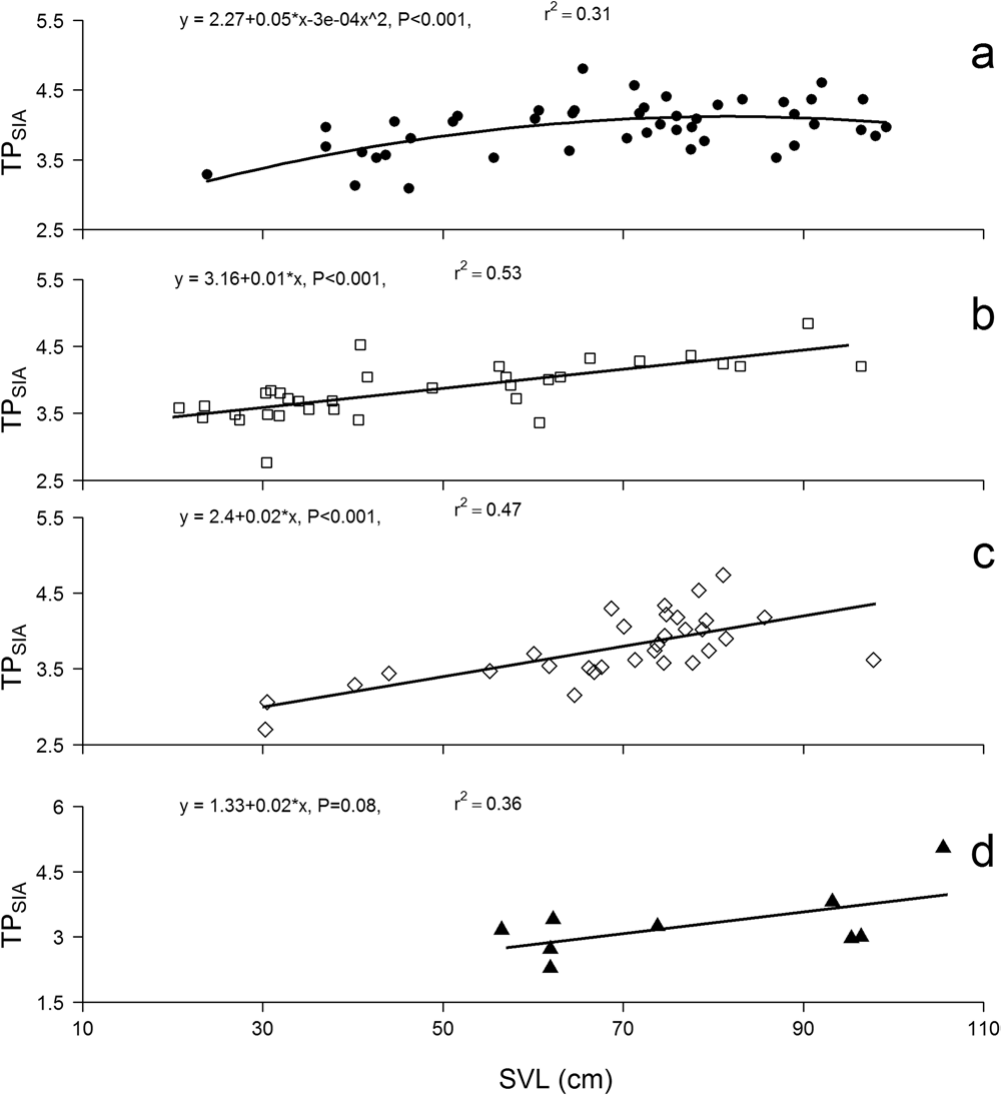
Ontogenetic shifts in *δ* ^15^N-derived estimates of trophic position (TP_SIA_) varying as a function of snout-vent length (SVL) in *Paleosuchus trigonatus* (**a**), *P*. *palpebrosus* (**b**), *Caiman crocodilus* (**c**) and *Melanosuchus niger* (**d**).

For all species studied here, we found significant positive relationships between TP_SIA_ and SVL (Fig. 1). The relationship between SVL and TP_SIA_ in *P*. *trigonatus* was best explained by a quadratic functional response, suggesting a plateau at maximum TP for mid-sized individuals (TP_SIA_ = 2.27 + 0.05*SVL − 0.0003*SVL^2^; F_2,42_ = 9.42; r^2^ = 0.31; p < 0.001). For the remaining three species, the relationship between SVL and TP_SIA_ was best explained by a linear functional response: *P*. *palpebrosus* (TP_SIA_ = 3.16 + 0.01*SVL; F_1,34_ = 38; r^2^ = 0.53; p < 0.001), *C*. *crocodilus* (TP_SIA_ = 2.39 + 0.02*SVL; F_1,29_ = 25.94; r^2^ = 0.47; p < 0.001), *M*. *niger* (TP_SIA_ = 1.33 + 0.02*SVL; F_1,7_ = 4.01; r^2^ = 0.36; p = 0.085).

**Figure 2:**
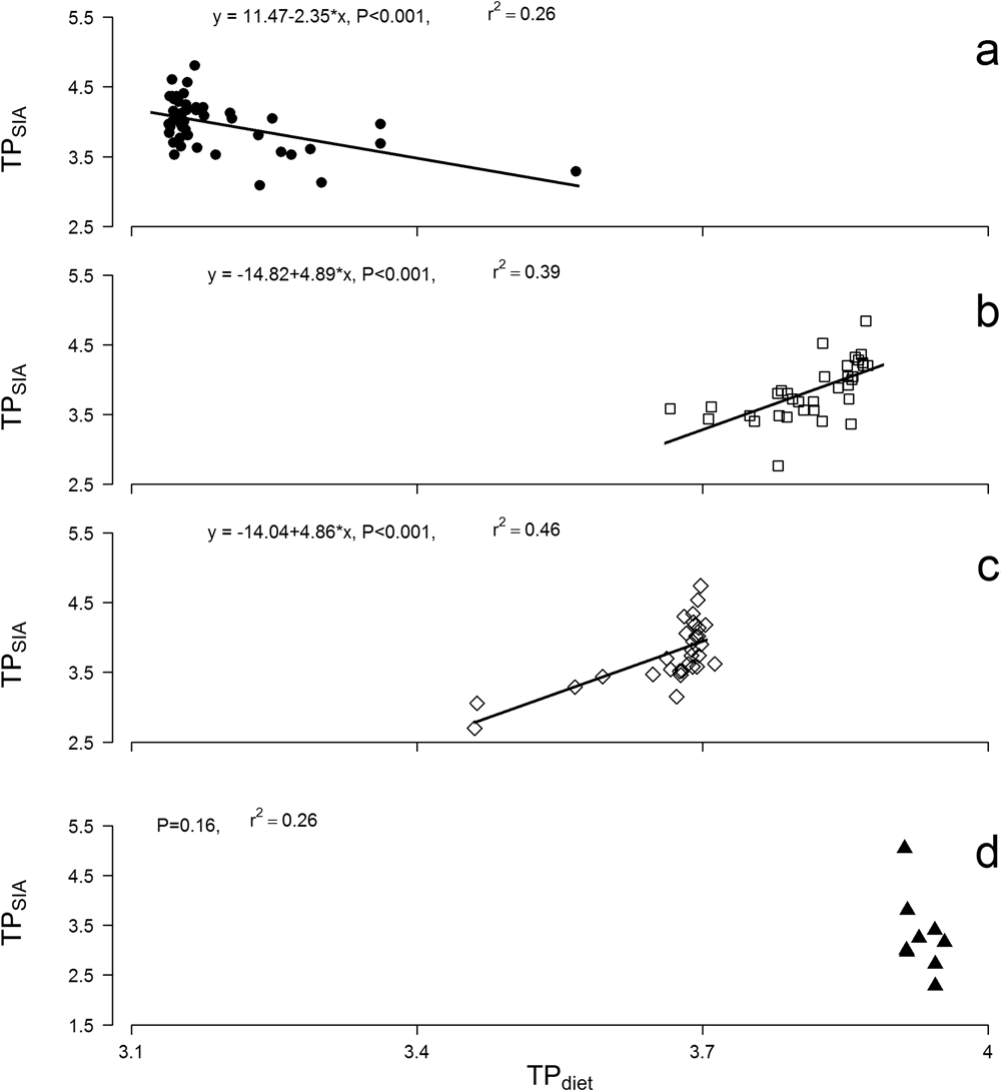
Ontogenetic shifts in *δ* ^15^N-derived estimates of trophic position (TP_SIA_) varying as a function of dietary-derived trophic position (TP_diet_) in *Paleosuchus trigonatus* (**a**), *P*. *palpebrosus* (**b**), *Caiman crocodilus* (**c**) and *Melanosuchus niger* (**d**).

Trophic position assessed using dietary data from the literature (TP_diet_) yielded different patterns than isotope-based trophic position. As a result, there was inconsistent agreement between TP_diet_ and TP_SIA_ (Fig. 2). In *P*. *trigonatus*, the relationship was significantly negative (TP_SIA_ = 11.47–2.35* TP_diet_; F_1,43_ = 15.27; r^2^ = 0.26; p < 0.001); whereas in *P*. *palpebrosus* (TP_SIA_ = −14.82 + 4.89* TP_diet_; F_1,34_ = 21.75; r^2^ = 0.39; p < 0.001) and *C*. *crocodilus* (TP_SIA_ = −14.04 + 4.86* TP_diet_; F_1,29_ = 24.43; r^2^ = 0.46; p < 0.001), the relationships were significantly positive. In *M*. *niger*, TP_SIA_ was not significantly influenced by TP_diet_ (p = 0.16; r^2^ = 0.26). TP_diet_ had much narrower ranges across body sizes within species and their trends were very different than those of TP_SIA_. Applying size-adjusted TDF values (see Methods), TP_SIA_*∆* values were more closely aligned with TP_diet_ in most of the cases. Overall TP_SIA_*∆* had narrower ranges across body sizes, and values in large individuals were more similar than TP_diet_ (Supplementary Fig. S1).

**Figure 3:**
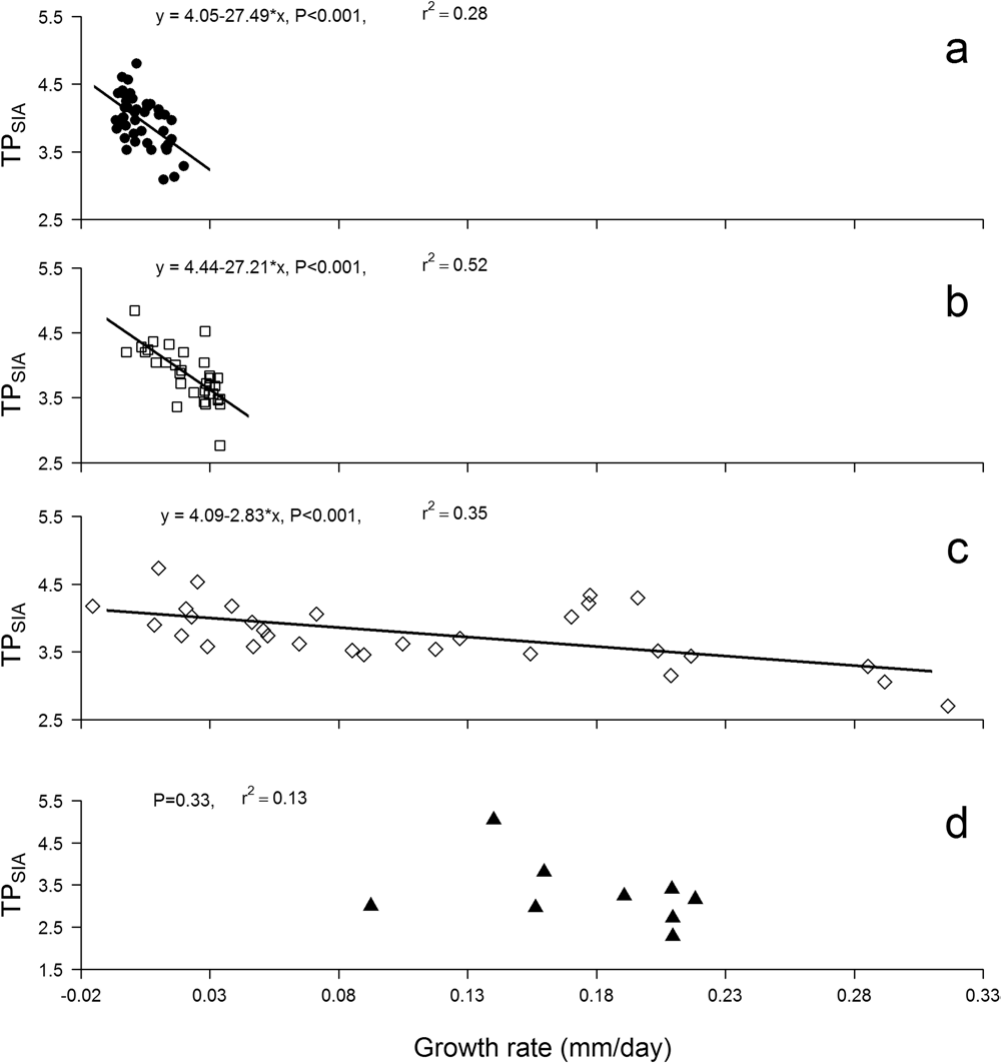
Ontogenetic shifts in *δ* ^15^N-derived estimates of trophic position (TP_SIA_) varying as a function of growth rate (GR) in *Paleosuchus trigonatus* (**a**), *P*. *palpebrosus* (**b**), *Caiman crocodilus* (**c**) and *Melanosuchus niger* (**d**).

Growth rates (GR) were negatively related to TP_SIA_ in *P*.*trigonatus* (TP_SIA_ = 4.06–27.49*GR; F_1,43_ = 18.11; r^2^ = 0.28; p < 0.001), *P*. *palpebrosus* (TP_SIA_ = 4.4–27.21*GR; F_1,34_ = 37.29; r^2^ = 0.51; p < 0.001) and *C*. *crocodilus* (TP_SIA_ = 4.09–2.83*GR; F_1,29_ = 15.32; r^2^ = 0.32; p < 0.001), but there was no significant relationship for *M*. *niger* (r^2^ = 0.13; p = 0.33) (Fig. 3).

**Figure 4:**
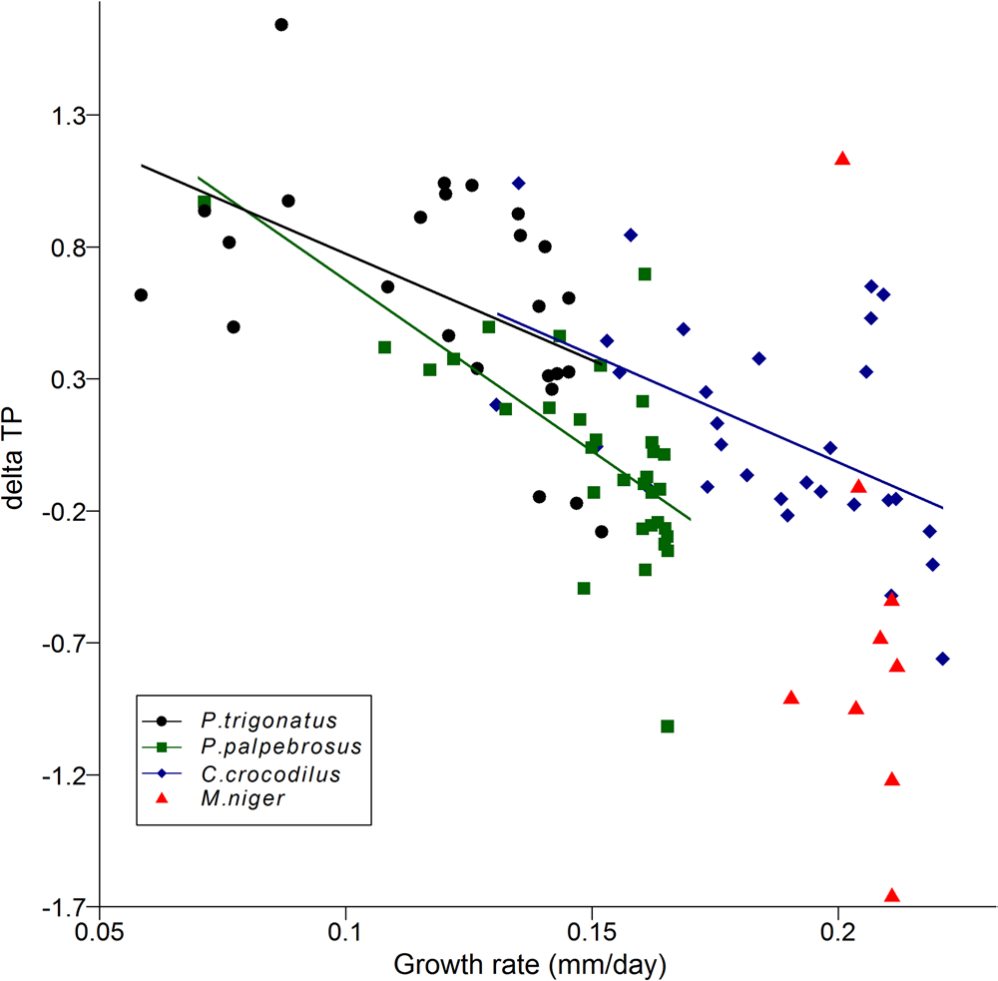
Intra- and interspecific variation in the difference between SIA- and diet-derived trophic position estimates (_delta_TP = TP_SIA_ − TP_diet_). _delta_TP varying as a function of growth rates in all four Amazon crocodilian species. Note log scale used on x-axis.

Similarly, when we analysed the influence of log-transformed growth rates on the difference between TP_SIA_ and TP_diet_ (_delta_TP), we found significant relationships in *P*. *trigonatus* (_delta_TP = −0.44–0.202*logGR; F_1,23_ = 8.04; r^2^ = 0.26; p = 0.009), *P*. *palpebrosus* (_delta_TP = −1.26–0.32*logGR; F_1,33_ = 29.7; r^2^ = 0.47; p < 0.001) and *C*. *crocodilus* (_delta_TP = −0.42–0.203*logGR; F_1,28_ = ; r^2^ = 0.26; p = 0.004), but not in *M*. *niger* (r^2^ = 0.09; p = 0.423; Fig. 4). When data from all species were combined, we found a significant negative relationship, accounting for nearly 40% of the variation, between log transformed growth rates and _delta_TP estimates (_delta_TP = −0.74–0.24 *log*GR; F_1,97_ = 64.02; r^2^ = 0.39; p < 0.001). Overall, isotope-based measurements underestimated TP in young, fast-growing individuals, and overestimated TP in old, slow-growing individuals (Supplementary Fig. S1).

### Models that best explained ontogenetic shifts in TP_SIA_

Our model selection process (AICc) indicated our top performing set of candidate models explaining variation in TP_SIA_ included models containing the main effects of growth rate and SVL singularly or both in combination with TP_diet_. Diet alone was insufficient to explain significant shifts in TP_SIA_ in any of the species (Table 1). After adjusting TDF values according to body size, the AICc selection procedure still indicated that the model using TP_diet_ alone was one of the worst models explaining variation in TP_SIA_∆ for three of the four species. Only in *C*. *crocodilus*, did body size-adjusted TDF values result in TP_diet_ being among the best models to explain TP_SIA_*∆* (Supplementary Table S2).

**Table 1:** Model selection process using Akaike’s Information Criterion (AICc) indicating sets of candidate models explaining variation in SIA-derived trophic position (TP_SIA_) of the four Amazonian crocodilian species. Abreviations are as follows: k = number of model parameters, AICc = Akaike’s Information Criterion, Δ AICc = Delta AICc, GR = growth rate, SVL = snout-vent length, TP_diet_ = dietary-derived trophic position.

Model rank	Species	Model structure	k	AICc	*Δ* AICc
1	*P*. *trigonatus*	GR	3	28.38	0.00
2		GR + SVL	4	29.22	0.84
3		TP_diet_ + GR	4	30.04	1.66
4		TP_diet_ + GR + SVL	5	30.50	2.12
5		TP_diet_	3	30.53	2.15
6		TP_diet_ + SVL	4	32.33	3.95
7		SVL	3	32.57	4.18
1	*P*. *palpebrosus*	SVL	3	16.38	0.00
2		GR	3	16.74	0.36
3		TP_diet_ + GR	4	17.13	0.75
4		GR + SVL	4	17.90	1.52
5		TP_diet_ + SVL	4	18.91	2.53
6		TP_diet_ + GR + SVL	5	19.84	3.46
7		TP_diet_	3	25.59	9.21
1	*C*. *crocodilus*	SVL	3	23.78	0.00
2		TP_diet_	3	24.64	0.87
3		TP_diet_ + SVL	4	25.67	1.89
4		GR + SVL	4	26.17	2.40
5		TP_diet_ + GR	4	26.59	2.81
6		TP_diet_ + GR + SVL	5	28.37	4.59
7		GR	3	30.44	6.66
1	*M*. *niger*	SVL	3	26.77	0.00
2		TP_diet_	3	28.13	1.36
3		GR	3	29.58	2.80
4		GR + SVL	4	31.70	4.93
5		TP_diet_ + SVL	4	32.01	5.23
6		TP_diet_ + GR	4	34.99	8.21
7		TP_diet_ + GR + SVL	5	41.36	14.59

## Discussion

Ontogenetic shifts in crocodilian trophic position revealed uncoupled trends between analyses based on *δ* ^15^N (TP_SIA_) and dietary information (TP_diet_). Overall, when derived solely using isotopic data, we found that trophic position estimates increased with increasing body size. Our findings using TP_SIA_ are in accordance with previous findings from other crocodilian species^14,34,35^. However, such increases in trophic position are not expected based on findings from stomach content analyses. Although most crocodilians show similar shifts in diet with increasing body size^44^, those changes relate to taxonomic categories of prey but not necessarily to shifts in prey occupying higher trophic levels. When we inferred trophic position of the Amazon crocodilians using literature-based dietary information, ontogenetic shifts in trophic position were minimal, and differed from those obtained when using stable isotope data. Although we based our dietary analyses on literature information obtained decades ago^28^, further dietary studies both within and outside the Amazon^32,34,45–48^ report similar ontogenetic dietary trends in Amazonian crocodilians. To our knowledge, ref.^28^ is the most detailed dietary analysis which includes data on prey quantities for all size classes of the four Amazonian crocodilians. We found that the estimated trophic position based on stable isotope analyses was much more likely to be related to physiological processes associated with size or growth rate than to diet composition for all four species we studied. This suggests that apparent ontogenetic shifts in trophic position based on isotope analyses in large ectothermic organisms, such as crocodilians, may be due to mechanisms other than dietary assimilation alone.

Some species of crocodilians may increase in mass by more than 10,000-fold throughout their lives, and as a result, are expected to experience ontogenetic dietary shifts. In general, young crocodilians consume terrestrial and aquatic invertebrates, shifting to more protein-rich diets (in terms of biomass) composed mostly of fish and terrestrial vertebrates as they grow larger^44^. According to traditional stomach-content analyses, the four species of Amazon crocodilians show subtly-different ontogenetic diet trajectories that lead to interspecific differences as adults. Juvenile *P*. *palpebrosus*, *C*. *crocodilus* and *M*. *niger* have diets mostly composed of terrestrial and aquatic invertebrates, changing gradually to fish as adults^28,32^. In contrast, *P*. *trigonatus* occurs in Amazonian closed-canopy headwater streams, where large fish are scarce^49^. Thus, juvenile *P*. *trigonatus* diet is mostly composed of terrestrial invertebrates, and ingestion of reptiles, amphibians and birds is more frequent in larger individuals. The latter prey items become less frequent in the largest size class, where terrestrial mammals become the most important prey in terms of volume for most individuals^28^. Although ontogenetic dietary shifts are universally found in crocodilians, they are not expected to result in consistent changes in trophic position for two reasons. Firstly, young crocodilians consume large amounts of both herbivorous and predatory invertebrates; the latter, such as Mygalomorph spiders, show high *δ* ^15^N values (F.Villamarín unpubl. SIA data). As a consequence, if dietary incorporation was the only mechanism determining the trophic position of crocodilians, young individuals should have an intermediate trophic position. Second, in diverse tropical freshwater food webs where there is a broad range of body sizes of primary consumers, a relationship between trophic position and body size across all taxa in the web is not expected^50^. Thus, even if crocodilians switch to a mostly piscivorous diet as they grow larger, we would not expect their trophic position to increase indefinitely because they may obtain enough dietary biomass by consuming large herbivorous-detritivorous fish, such as common species of Characidae, Loricariidae and Prochilodontidae. Overall, the *δ* ^15^N values of prey from aquatic and terrestrial sources in our study was unrelated to body size (Supplementary Fig. S3).

Ontogenetic dietary shifts have traditionally been assumed to be the main mechanism influencing shifts in isotope-based estimates of trophic position in crocodilians^14,34,35^. However, if diet alone was responsible for trends in nitrogen stable isotopes in crocodilians, estimates of trophic position are not expected to increase linearly with body size for the reasons given above. Estimates of the trophic position of an organism based on *δ* ^15^N depend on various factors including the baseline organism used^4,51^, cross-food web feeding^14^, the isotopic composition of its diet and the degree of isotopic discrimination occurring between trophic levels^4–7^. While the former two factors are relatively easier to account for, the latter two may be more difficult to determine with sufficient precision to estimate trophic position, and complimentary direct studies of behavior or gut contents are needed for some species^36,52,53^. Thus, low concordance between consumer *δ* ^15^N-derived estimates of trophic position and stomach-content analysis is not uncommon in other organisms^36,52,53^. We therefore argue that other size-related mechanisms involved are important in determining trends in stable isotope values of crocodilians.

Discrimination of *δ* ^15^N is believed to be affected by rates of N excretion relative to assimilation^11^. If assimilation efficiency is ontogeny dependent, then discrimination is likely to be proportional to growth rate^39,54,55^. This may not be a serious problem for many organisms because they belong to short food chains and individuals vary little in size. However, for many species of ectothermic vertebrates, foraging individuals may vary in size by several orders of magnitude, and growth rates are generally closely related to size^23,37,38^. In this case, discrimination, and hence trophic position estimation, cannot be evaluated without taking size into account. This is especially critical for studies aimed at evaluating the relationship between trophic position and body size. The relationship between body size and TP_SIA_ estimates in our analyses were fairly scattered, thus other factors besides body size might be responsible for individual variation^56^ in trophic position, leading to unexplained variation in this relationship.

There are other possible dietary explanations that could drive shifts in estimates of trophic position of crocodilians based on *δ* ^15^N. First, the overestimation of trophic position for large individuals may be due simply to slow isotopic turnover. Animals with large body sizes have tissues with the longest isotopic half-lives^57,58^, and crocodilians have particularly slow turnover rates^16^. Therefore, shifts to low-trophic position prey by large crocodilians may be barely discernible in their tissue *δ* ^15^N values. Indeed, we found evidence of the highest *δ* ^15^N values in medium-sized *P*. *trigonatus*, a pattern similar to that observed elsewhere^14^ for *Crocodylus porosus*, but all other species were best fit by linear regressions. The largest individuals of *P*. *trigonatus* eat mainly herbivorous mammals, so the small reduction in apparent trophic level may be a consequence of diet, even though there is still a strong legacy from former prey. Of the four species we studied, the only one for which we did not have an adequate sample of large individuals was *M*. *niger*. This species can attain total lengths of over 5 m. Past overhunting of this species caused a severe reduction in its density and, although populations are currently increasing, we could not obtain samples of very large-sized individuals due to their wariness and low densities in the study area (F.Villamarín pers. obs.).

An additional dietary factor that may influence TDF and, thus, estimation of trophic position, is diet quality^59^. High discrimination can occur from either low protein quality or high protein content in the diet. If the protein content of crocodilian diets increases as they age, we could expect greater discrimination in older individuals. Generally speaking, as obligate predators, crocodilians have a diet that is high in protein throughout their lifespan, so this is unlikely to be as strong a factor as would be expected for omnivorous animals. An increasing trend in TDF values from 2.5 to 3.4‰ as crocodilians age was simulated in our analyses to examine the sensitivity of the trophic position estimate using varying TDF values^39^. Even after adjusting the values to maximally support the relationship between estimated trophic position based on isotopes and diet, the AICc selection procedure still indicated that the model using TP_diet_ was one of the worst to explain TP_SIA_ estimates for three of the four species. Only in *C*. *crocodilus*, did increasing TDF values result in TP_diet_ being among the best models to explain TP_SIA_. This highlights the sensitivity of estimates of TP_SIA_ to estimates of TDF values^4,5,52,60,61^. In the absence of a strong physiological model to explain the ontogenetic change in TDF, such adjustments are purely *ad hoc* and cannot be used to further our understanding of ontogenetic shifts in crocodilian trophic position.

Not all species show similar TDF values; those with diets high in protein show higher TDF values^11,59^. Independent of the diet, some species of crocodilians, show much lower discrimination values than other vertebrates^15,16^. This is crucial, because the importance of growth-related factors will be greater in relation to food-related factors for these species. Isotopic discrimination is the most sensitive parameter in trophic position estimates because TDF values enter the denominator of the equation to calculate trophic position^4^. Therefore, any reduction in TDF values causes significant increases in estimates of trophic position. If TDF values are ontogeny-dependent in crocodilians, then growth-rate-related TDF values might be causing the apparent increases in trophic position that are not directly related to diet. The clear negative influence of growth rates on the difference between SIA- and dietary-derived TP both intra- and inter-specifically is strong evidence that values of *δ* ^15^N in tissues of Amazonian crocodilians principally reflect their growth rates or some other physiological factor related to size. This conclusion is likely to apply to many other large, long-lived ectothermic vertebrates.

One of the key parameters to calculate trophic position is an appropriate *δ* ^15^N baseline value^4,51^. Baseline *δ* ^15^N values must be able to integrate temporal and spatial isotopic changes to adequately reflect those of larger consumers. Thus, long-lived herbivores have commonly been used as baselines in foodweb studies^4,51,62^. We used mean *δ* ^15^N values of available primary consumers as the system’s aquatic and terrestrial baselines^51,63^. This approach typically yields results in which baseline variation is not a major source of error in trophic position estimates^63^. Furthermore, if consumers ingest food from a number of distinct food chains, such as species that use aquatic and terrestrial food resources, supplementary information on the proportional contributions of those sources to the diet, such as that provided by *δ* ^13^C data, is necessary to estimate trophic position based on *δ* ^15^N. After correcting for this source of uncertainty by applying the proportional contributions of aquatic and terrestrial carbon resources to the crocodilian tissues^19^ to calculate trophic position, we were confident of accounting for habitat variability in *δ* ^15^N sources.

The considerations we have presented in relation to crocodilians are likely to apply also to their prey, many of which are also ectotherms with large variation in size and ontogenetic variation in growth rates. Isotopic discrimination by prey does not only depend on their growth rate, it also depends on the proportions of different tissues, such as chitin or muscle. Therefore, stable isotope estimates of trophic position for generalist predators may not clearly reflect the trophic positions of their prey. In this case, interpretation of isotope values would require detailed information on diet, but such data would often make the isotope estimates redundant. Controlled experiments to estimate TDF values in a broad range of crocodilian size classes fed the same diet may help to better understand ontogenetic shifts in *δ* ^15^N and test the hypotheses presented here, but direct observations on diet are likely to remain better indicators of trophic position in wild crocodilians than estimates of trophic position based on stable isotopes.

## Methods

### Study region

This study was conducted in lotic waterbodies, including most habitat types where crocodilians occur in the Central Amazon region, within the limits of the Piagaçu-Purus Sustainable Development Reserve and the interfluvium between the Madeira and Purus Rivers. A detailed description of the study area can be found in ref.^19^.

### Sampling of baseline organisms

Primary consumers were collected at each study site to characterize isotope values at the base of the food web. Aquatic molluscs (*Pomacea* sp. snails) were captured by hand according to their availability and used as the baseline aquatic end-member. Claw samples from terrestrial herbivorous vertebrates, such as agouti (*Dasyprocta sp*.) and paca (*Cuniculus sp*.), were obtained opportunistically from subsistence hunters in the area. These values were used as the baseline for the terrestrial-prey source pathway as they were not significantly different from *δ* ^15^N values of terrestrial invertebrates such as Formicidae, Passalidae and Scarabaeidae (T-test; t = −1.67; df = 1.1; p = 0.327). All isotope samples collected were kept frozen in liquid nitrogen for approximately one month before their return to the laboratory.

### Crocodilian sampling

Crocodilians were captured using fyke nets in headwater streams and steel snares at night in other water bodies. After measuring (snout-vent length, SVL), sexing and weighing the animals, a piece of claw and a small piece of dorsal tail muscle underlying the scutes were removed and rinsed with distilled water to avoid contaminating the sample with blood. We used claw or muscle-tissue samples for analyses, depending on the highest number of collected samples per species. Inter-tissue variability in crocodilians is minimal^14^. Since muscle tissue shows a slow isotopic turnover rate and claws are inert tissues which accumulate isotopic values throughout the consumer’s life span, both tissues reflect long-term isotopic values of crocodilians.

All research procedures were conducted in accordance with all applicable laws and rules set forth by the Brazilian government and involved institutions, and all necessary permits were in hand when the research was conducted. Collecting permits were issued by ICMBio-SISBIO No. 28648-1, 28648-2, 28648-3, 28648-4. Ethical approvals for handling animals were issued by *Comissão de Ética em Pesquisa no Uso de Animais* (CEUA-INPA), No. 024/2013.

### SIA Laboratory processing

All samples were kept frozen at −20 °C in the laboratory. They were dried in an oven at 60 °C for 24 to 48 h before grinding and homogenizing with a mortar and pestle. Samples were combusted in a EuroEA 3000 (EuroVector, Italy) or Europa GSL (Sercon Ltd, Crewe, UK) elemental analyzer and the resulting N_2_ gases were chromatographically separated and fed into an IsoPrime (Micromass, UK) or Hydra 20–22 (Sercon Ltd, Crewe UK) isotope-ratio mass spectrometer. These measure the ratio of heavy and light isotopes in a sample relative to an international standard. Isotope ratios (*δ*) are expressed in parts per mil (‰), defined as *δ* (‰) = (R_sample_/R_standard_ − 1) * 1000, where R_sample_ and R_standard_ are the isotope ratios of the sample and standard, respectively. Isotopic standards were referenced to atmospheric air for nitrogen. Mean *δ* ^15^N ± SD difference between repeated measurements of samples was 0.18 ± 0.02‰.

### Statistical analysis

#### Methods to calculate trophic position of crocodilians

***δ*** ^**15**^**N-derived trophic position of crocodilians** (**TP**_**SIA**_) **using a fixed TDF value**: We calculated TP_SIA_ of crocodilians based on the following equation modified from Post (2002):

1 $$TP=\lambda +({\delta }^{15}N{.}_{croc}-[{\delta }^{15}N.{}_{terr.base}\,\ast \,\alpha +{\delta }^{15}N.{}_{aq.base}\ast (1-\alpha )])/{{\rm{\Delta }}}^{15}N$$

where *δ* ^15^*N*._*croc*_ is the nitrogen stable isotope value of crocodilian tissues; *δ* ^15^*N*._*terr*.*base*_ and *δ* ^15^*N*._*aq*.*base*_ are nitrogen stable isotope values of terrestrial and aquatic baselines (4.6‰ and 3.8‰, respectively); α is the proportional contribution of carbon from terrestrial origin (obtained from ref.^19^); λ is the trophic level of the organisms used to estimate *δ* ^15^*N*._*terr*.*base*_ and *δ* ^15^*N*._*aq*.*base*_ (e.g., λ = 2 for primary consumers); and *Δ* ^15^N is the TDF, or trophic enrichment in *δ* ^15^N per trophic level.

TDF of ^15^N in crocodilians (around 1.0‰^15,16^ can be much lower than in most organisms studied (3.4‰^4,6^). Thus, we used a conservative *Δ* ^15^N value of 2.5‰ for crocodilians which is a mean TDF value also reported elsewhere^61^.

***δ*** ^**15**^**N-derived trophic position of crocodilians** (**TP**_**SIA**_***∆***) **using variable TDF values**: In order to understand sensitivity of trophic position estimates using different TDF values (*Δ* ^15^N), an increasing trend in TDF from 2.5 to 3.4‰ as the crocodilians age was simulated in our analyses. This ~1‰ change is in the range of observed TDF values for animals with variable growth rates^39,64^. For this purpose, we assigned a value of 2.5‰ to the smallest individual and 3.4‰ for the largest. We then used a linear regression to predict individual *Δ* ^15^N values according to crocodilian SVL. Finally, we applied length-specific *Δ* ^15^N values for each individual to calculate trophic position using equation (1).

**Dietary trophic position of crocodilians** (**TP**_**diet**_): We used the equation proposed by ref.^65^ to calculate the expected trophic position of crocodilians based on their diet:

2 $$TL=1+(\sum _{j=1}^{n}Pj\,\ast \,TLj)$$

where *P*_*j*_ is the proportion of each prey category *j* in the diet of the predator, *TL*_*j*_ is the trophic level of each prey category *j* and *n* is the total number of prey species in the diet.

- Based on trophic habits, *TL* values of prey items were assigned as follows: Herbivores = 2, omnivores = 2.5, carnivores = 3.

To calculate *P*_*j*_:

- Published information on the mean number of prey individuals consumed per crocodilian per size class was obtained from Reference 28. We ran linear regressions based on the relationship between the mean number of prey per crocodilian against their snout-vent length (SVL) to estimate the expected number of each prey category per crocodilian of a given size (*Prey.croc*).
- We obtained mean mass values of prey from the literature^66^ - mammals), personal communications (Rafael de Fraga - snakes) and this study (all other organisms). Based on the mass of a prey item that a crocodilian of 100 cm SVL would ingest (which would be the largest possible size of the prey), we calculated the expected mass (*Prey.mass.exp*) of a prey item that a crocodilian of a given volume (SVL^3^) would ingest.
  3 $$Prey.mass.exp=Prey.mass\,\ast \,SV{L}^{3}/{100}^{3}$$
- We estimated prey mass per crocodilian:
  4 $$Prey.mass.croc=Prey.croc\,\ast \,Prey.mass.exp$$
- To calculate Pj, we estimated the proportional contribution of each prey category within the diet of that crocodilian species based on Prey.mass.croc and aquatic (Aq.prop) vs. terrestrial (Terr.prop) contributions derived from δ ^13^C^19^:

5 $$\begin{array}{ccl}PjTerr & = & Prey.mass.croc.Terr\ast Terr.prop/\\  &  & (\sum Prey.mass.croc.Terr\ast Terr.prop\\  &  & +\,\sum Prey.mass.croc.Aq\ast Aq.prop)\ast 100\end{array}$$

6 $$\begin{array}{ccl}PjAq & = & Prey.mass.croc.Aq\ast Aq.prop/\\  &  & (\sum Prey.mass.croc.Terr\ast Terr.prop\\  &  & +\,\sum Prey.mass.croc.Aq\ast Aq.prop)\ast 100\end{array}$$

### Growth rates

We estimated growth rates of the four species based on their size, using equations published in the literature (*P*. *trigonatus*^42^, *P*. *palpebrosus*^40^, *C*. *crocodilus*^41^ and *M*. *niger*^43^). Growth rates in those studies were calculated using mark-recapture procedures in wild populations of all four species over periods between six and 10 years. The growth rates (mm/day) were estimated as the difference in SVL between captures divided by the interval between recaptures.

### Akaike Information Criteria

We used simple regressions to evaluate how *δ* ^15^N-derived trophic-position values (TP_SIA_) change as a function of snout-vent length (SVL), dietary-derived trophic position (TP_diet_) and growth rates (GR), independently. Additionally, we used multiple regressions with a combination of those independent variables and used the Akaike Information Criteria (AICc) to evaluate which models best explained the changes in TP_SIA_.

In order to examine the sensitivity of the estimation of trophic position using varying TDF values, we used TP_SIA_*∆* as a dependent variable. We then used AICc selection procedure to evaluate which model best explained changes in trophic position derived using different TDF values as was done for the analyses with constant TDF.

Finally, we explored influences of growth rate on both dietary- and *δ* ^15^N-derived estimates of trophic position of crocodilians within and among species. We first calculated the difference between TP_diet_ and TP_SIA_ (_delta_TP) for each individual of each species. We then used a simple regression, both specifically or pooling all species together and graphed _delta_TP against log-transformed growth rates. Based on the work of ref.^39^, we expected _delta_TP to decline with increasing growth rate.

All statistical analyses and graphics were run using R software^67^. The datasets generated during the current study are available in the Research Program on Biodiversity (PPBio) repository, https://ppbio.inpa.gov.br/repositorio/dados.

## Electronic supplementary material

Supplementary material
