# Supplementary material for: Body size is more important than diet in determining stable-isotope estimates of trophic position in crocodilians
Source: Sci Rep. 2018 Jan 31;8:2020. doi: 10.1038/s41598-018-19918-6 (PMC5792559; doi:10.1038/s41598-018-19918-6)
Supplement: Supplementary file 1 — Supplementary material [file 41598_2018_19918_MOESM1_ESM.pdf]

Supplementary material

Body size is more important than diet in determining trophic position of crocodilians.

Villamarín, F.; Jardine,T.D.; Bunn, S.E.; Marioni, B. and Magnusson, W.E. *Scientific Reports.*

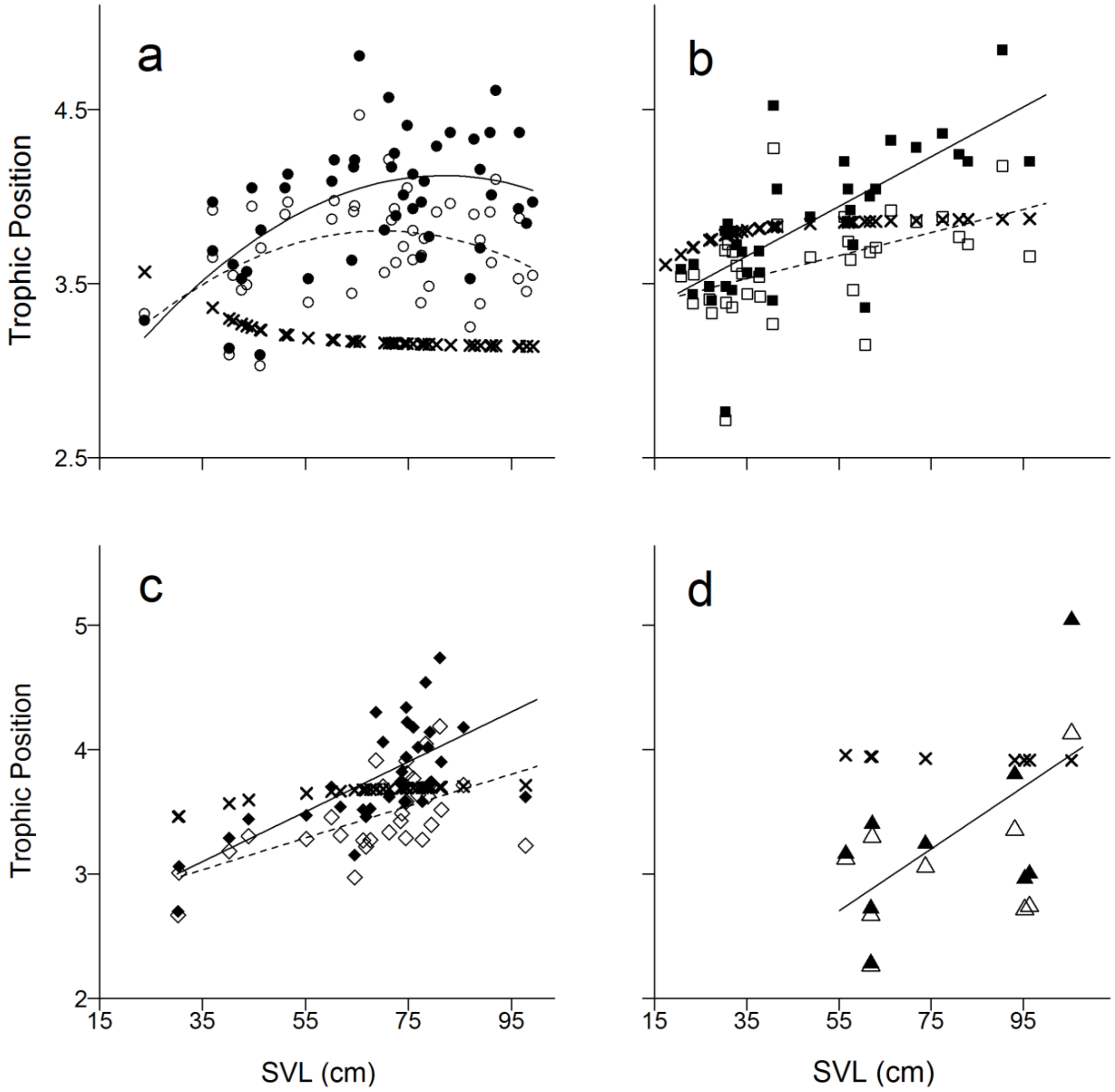

Supplementary Figure S1 online. Trends in trophic position of a) *Paleosuchus trigonatus*, b) *P. palpebrosus*, c) *Caiman crocodilus* and d) *Melanosuchus niger*, as a function of body size (snout-vent length; SVL). Trophic position was assessed using three different approaches: δ^{15}N-derived trophic position using a fixed trophic discrimination value of 2.5‰ (TP_{SIA}; filled symbols with continuous line); δ^{15}N-derived trophic position using increasing trophic discrimination values ($TP_{SIA}\Delta$; hollow symbols and dashed line); and dietary-based trophic position (TP_{diet}; cross symbols).

Supplementary Table S2 online. Model selection process using Akaike´s Information Criterion (AICc) indicating sets of candidate models explaining variation in SIA-derived trophic position calculated using body size-adjusted isotopic discrimination values ($TP_{SIA\Delta}$) of the four Amazonian crocodilian species. Abreviations are as follows: **k** = number of model parameters, **AICc** = Akaike´s Information Criterion, ***Δ*AICc** = Delta AICc, **GR** = growth rate, **SVL** = snout-vent length, $\mathbf{TP_{diet}}$ = dietary-derived trophic position

Model rank	Species	Model structure	k	AICc	ΔAICc
1		GR+SVL	4	17.77	0.00
2		TP_{diet}+GR+SVL	5	18.78	1.01
3		TP_{diet}	3	18.79	1.03
4	*P. trigonatus*	GR	3	19.39	1.62
5		TP_{diet}+SVL	4	20.54	2.78
6		TP_{diet}+GR	4	21.13	3.36
7		SVL	3	21.14	3.37
1		GR	3	6.96	0.00
2		SVL	3	7.91	0.96
3		TP_{diet}+GR	4	8.90	1.95
4	*P. palpebrosus*	GR+SVL	4	9.47	2.51
5		TP_{diet}	3	10.10	3.14
6		TP_{diet}+SVL	4	10.39	3.43
7		TP_{diet}+GR+SVL	5	11.03	4.07
1		TP_{diet}	3	10.70	0.00
2		SVL	3	11.58	0.88
3		TP_{diet}+SVL	4	12.98	2.28
4	*C. crocodilus*	TP_{diet}+GR	4	13.03	2.34
5		GR+SVL	4	13.99	3.29
6		GR	3	15.41	4.71
7		TP_{diet}+GR+SVL	5	15.71	5.02
1		SVL	3	22.07	0.00
2		TP_{diet}	3	22.85	0.79
3		GR	3	23.64	1.57
4	*M. niger*	GR+SVL	4	27.35	5.28
5		TP_{diet}+SVL	4	27.65	5.58
6		TP_{diet}+GR	4	29.68	7.61
7		TP_{diet}+GR+SVL	5	37.51	15.44

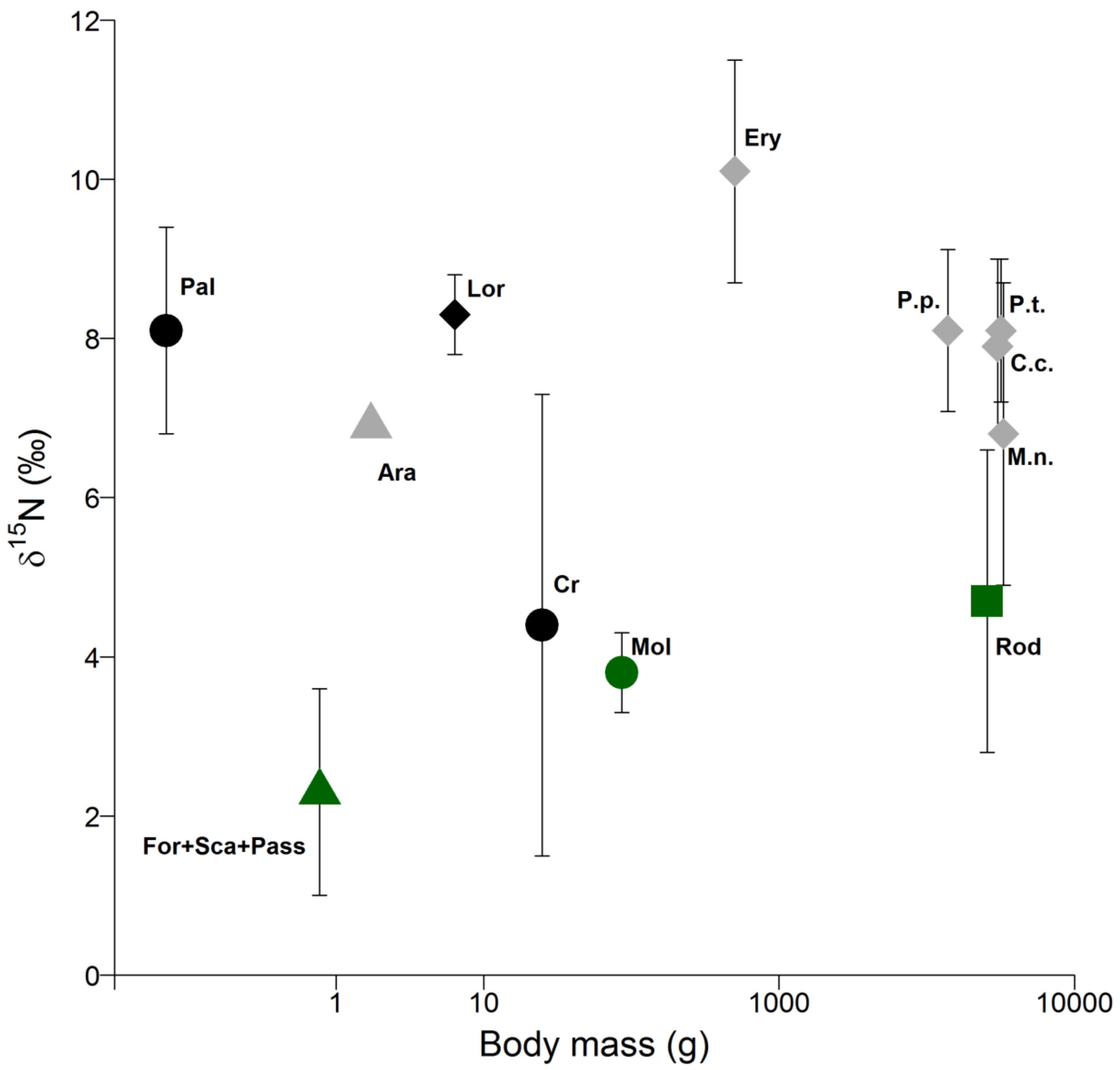

Supplementary Figure S3 online. Mean ± SD of δ^{15}N values as a function of body mass (log-transformed) of consumers: Terrestrial Invertebrates (triangles), Aquatic invertebrates (circles), Terrestrial vertebrates (squares), Aquatic vertebrates (diamonds). Colours represent trophic groups: Herbivores (green), Omnivores (black) and Carnivores (gray). Abbreviations of organisms are as follows: **Sca**=Scarabaeidae; **For**=Formicidae; **Sca**= Scarabaeidae; **Pass=** Passalidae; **Cr**=crab (Trichodactylidae); **Ara**=Araneae (Mygalomporphae); **Pal**=Palaemonidae ; **Lor**=Loricariidae; **Mol**=mollusc (Ampullariidae); **Ery**=Erythrinidae; **Rod**=Rodentia; **P.t.**=*Paleosuchus trigonatus*, **P.p.**=*P.palpebrosus*, **C.c.**=*Caiman crocodilus*, **M.n.**=*Melanosuchus niger*. Note log scale used on x-axis.
